# Good Asylum Attendants for the Insane

**Published:** 1886-10-23

**Authors:** 


### GOOD ASYLUM ATTENDANTS FOR THE INSANE.

[Communications for this Column should be addressed, "Superintendent," care of the Editor of THE HOSPITAL.]

**Of Interest to All.** THE question of the successful selection, training, and retention of the services of good asylum attendants must be a matter of interest and importance to all classes. To those who have unfortunately to provide for the care of the mentally afflicted in private houses, this question is of permanent importance and difficulty. Although such attendance is now one of the recognised branches of nursing, it is very difficult to secure a good attendant whose services are of incalculable value in the home treatment of a case of insanity. Through such services attacks may be cut short, infinite anxiety and risks may be saved to the patients and relatives, accidents may be avoided, suicides averted, and valuable lives restored to reason. To the asylum superintendents, and to the inmates of public institutions, all such considerations have an importance, which it is probably impossible to exaggerate. To the general public, and especially to the younger members of both sexes belonging to the classes from which asylum attendants are drawn, this branch of the labour market, its opportunities and its prospects, cannot fail to be of interest. In these circumstances we shall make no apology for bringing the whole question under the consideration of our readers, in the belief that those who require the services of good attendants, whether in private families or public institutions, and that those who either are or who may wish to become good attendants themselves, will welcome a plain statement of the facts and possibilities of the situation at the present time.

**Published Facts.** In consequence of the announcement we made of our intention to publish an article on the training of asylum attendants, male and female, we have received several communications on the subject. It appears that the question has been dealt with by various writers during recent years, in the *Quarterly Journal of Mental Science* (edited by Dr. Hack Tuke) by Dr. Clouston and Dr.

Shuttleworth, amongst others. A sub-committee of the Medical Psychological Association has published a hand-book for the instruction of attendants on the insane (Baillière and Co., London), which seeks to give simple notions of the body and mind in health and disease, instructions for the management of maladies with which attendants are usually brought into contact, and rules for their guidance in matters of every-day experience. The most recent and exhaustive publication has been issued, however, by the State Charities Aid Association, New York. This report is based upon material supplied by the superintendents of American and British asylums, and constitutes a most valuable monograph on the whole question. It has been thought desirable to mention these various publications, to enable those interested, to refer to previous writers on the subject. It is, besides, an act of justice to do so, because the articles which we propose to devote to this question will be based to a considerable extent upon the facts contained in the publications of the gentlemen here mentioned.

**Fifty Years Ago and Since.** An able American writer has truly said that during the last fifty years great progress has been made in the treatment of insanity. At the beginning of this century lunatics were generally regarded as dangerous wild beasts, to be caged, chained, and cowed into submission for the protection of society. They are now considered as sick people, to be cured if possible, and to receive during the continuance of their malady every practicable alleviation of their sufferings. In pursuance of this object there has been, in all the best managed asylums, a gradual substitution of personal supervision and attendance for mechanical methods. The patients now enjoy a degree of freedom (notably in some of the Scotch asylums) which would formerly have been thought incompatible with safety. The new system naturally demands of the attendants, both men and women, to whom the immediate care of the insane has been entrusted, qualifications much higher than were formerly thought necessary. Indeed, the difficulty of procuring and retaining persons fitted to perform the onerous duties of such a position is probably the gravest of all those with which the superintendents in insane asylums have to contend. The ordinary asylum attendant of the old school regarded the patients merely as inmates and himself as a keeper; his highest ideal was the preservation of order; of curative measures, of the study of the individual peculiarities of the patient, of intelligent effort to meet the ever-varying needs of each case, he had not the remotest conception. Intentional brutality and neglect are probably rare (although the study of lunacy reports shows that they are by no means unknown), ignorance is lamentably common. The impression such facts have upon those engaged in nursing the sick outside asylums, as well as upon the attendants at asylums, will be realised by the statement of this undoubted fact. The ablest superintendents of the best known training-schools for nurses often object to receive for training women who have been employed in asylums, because they declare them as a class to have wrong ideas of the relation between nurse and patient, and, in consequence, their manner to sick people is always rough and generally dictatorial.

**The Ideal Attendant.** Dr. Clouston states that he should not much exaggerate if he said that this attendant question is, at present, the question of questions, to many superintendents at the head of asylums. What superintendent would not feel a burden lifted off, and would not sleep more soundly at night if he could think that his patients were all under the care of experienced, intelligent, and trustworthy attendants? The absence of that suspicion, with which they have instinctively to go round their wards, would sweeten their lives and liberate more energy and sympathy in doing the daily medical work. If they could see in each of their attendants a well-principled person, intelligent enough to understand the reason of his rules and the un-reason of his patients; with sense of duty enough to make him do his work as well when his superior officers are away as when they are looking on; with vigour of mind enough to compel the respect of his fellow-attendants and the patients; with tact and temper enough to get on smoothly and to have his own way with them too; with kindness of heart enough to put himself in the position of the patients at times; with self-control enough never to do more than blow some of them up, when they needed it; with observation enough to see and report the changes in their mental and bodily state to the doctor; with adaptability enough to cheer up the depressed, and to curb the excited in the same breath; and finally, with physique and health strong, with a gracious presence and a pleasant, sympathetic manner. If each superintendent could see all this embodied in each of his attendants he would feel as if it were an easy thing to manage an asylum, and not so sad a thing after all to be insane.

**The Present Want.** An attempt to realise the ideal asylum with such attendants is rightly regarded by Dr. Clouston to be, indeed, one of the highest embodiments of humanity, philanthropy, and care. This ideal may be reached in all asylums, and has already been nearly reached in some, since Dr. Clouston wrote this word-picture in October 1876. There are at present to be found in most asylums some such ideal attendants at any rate, and a tendency is setting in the direction of the speedy realisation of what was but an ideal picture ten years ago. What is still undoubtedly wanted is not only more material to select from, but an awakening of the public conscience to the wide field of usefulness that lies open to workers, as nurses and attendants in asylums, and the introduction of a proper system of training for good asylum attendants, throughout the English-speaking world. (*To be continued.*)

